# Changes in Human Milk Immunoglobulin Profile During Prolonged Lactation

**DOI:** 10.3389/fped.2020.00428

**Published:** 2020-08-07

**Authors:** Matylda Czosnykowska-Łukacka, Jolanta Lis-Kuberka, Barbara Królak-Olejnik, Magdalena Orczyk-Pawiłowicz

**Affiliations:** ^1^Department of Neonatology, Wroclaw Medical University, Wrocław, Poland; ^2^Department of Chemistry and Immunochemistry, Wroclaw Medical University, Wrocław, Poland

**Keywords:** breastfeeding, immunoglobulins, prolonged lactation, child nutrition, human milk

## Abstract

Mother's milk immunoglobulins (Igs) delivered to infants during breastfeeding are crucial in shaping and modulating immature infants' immune system and provide efficient protection against pathogens. The aim of the study was to evaluate the immunoglobulin concentrations in milk of 116 lactating mothers over prolonged lactation from the 1st to the 48th month using the ELISA method. The concentration of proteins, SIgA and IgG, but not IgM, showed a positive correlation (*r* = 0.69, *p* < 0.005; *r* = 0.54, *p* < 0.05; and *r* = 0.27, *p* < 0.05, respectively) with lactation from the 1st to the 48th month. The lowest concentrations of SIgA and IgG were observed for the first year (2.12 ± 0.62 g/L and 14.71 ± 6.18 mg/L, respectively) and the highest after the 2nd year of lactation (7.55 ± 7.16 g/L and 18.95 ± 6.76 mg/L, respectively). The IgM concentration remained stable during 2 years (2.81 ± 2.74 mg/L), but after 24 months it was higher (3.82 ± 3.05 mg/L), although not significantly. Moreover, negative correlations of protein (*r* = −0.24, *p* < 0.05) and SIgA (*r* = −0.47, *p* < 0.05) concentrations with the number of feedings were found. Human milk after the 2nd year of lactation contains significantly higher concentrations of protein, SIgA, and IgG. High concentration of immunoglobulins and protein during prolonged lactation is an additional argument to support breastfeeding even after introducing solid foods and should be one of the overarching goals in the protection of children's health.

## Introduction

During breastfeeding, the mother's milk delivers to the immunologically immature newborn and infant both elements of the immune system, namely, adaptive, and innate immune components. In contrast to the non-specific innate defense, the adaptive immune protection is highly specific and characterized by memory to pathogens to which there was a previous exposure (1, 2). According to Brandtzaeg (3, 4), the content of milk secretory immunoglobulin A (SIgA) generally reflects the antigenic stimulation of the mother's mucosal immune system by intestinal (gut-associated lymphoid tissue) and respiratory pathogens (nasopharynx-associated lymphoid tissue). In light of the cited information above, mother's milk immunoglobulins are very important players in shaping and modulating the maturation of the newborn's immune system and provide efficient protection against pathogens (5–7). This phenomenon is particularly relevant since, at an early stage of life, newborns do not yet produce their own repertoire of immunoglobulins (8) and use immunoglobulin G (IgG), which was transferred from the mother's circulation through the placenta. However, the immunoglobulin resources can be replenished by immunoglobulins with mother's milk if children are fed naturally.

The most abundant immunoglobulin in human milk is SIgA, which represents over 90% of milk antibodies. However, immunoglobulins G and M (IgM) are also present, but in concentrations much lower than SIgA (9–13). The immunological profile of mother's milk is dynamic and can be influenced by a wide range of factors, among which week of the gestation and lactation period, namely, milk maturation, are the most frequently assessed parameters in this respect.

The highest concentration of SIgA is reported for early colostrum (days 2–5, ~2.5 g/L), and then the level is relatively stable throughout transitional (days 8–12, ~1 g/L) to mature milk (days 26–30, ~0.7 g/L) (14). On the other hand, the latest study demonstrated that over-lactation of mothers who gave birth to full-term infants (>37 weeks of gestation) from the 2nd day to the 24th week of lactation resulted in the concentration of SIgA being higher at the beginning of lactation and then gradually decreasing until 6 months (15). Over the first year of lactation, the SIgA concentration was significantly lower than for the first month (16). However, from the 11th to the 17th month of lactation, an upward trend of SIgA was found, from 0.21 to 0.29 g/L, respectively (16). However, according to Trend, the concentration of SIgA during the 1st month of lactation does not differ significantly between gestation groups (term and preterm birth), regardless of the degree of prematurity, namely, moderately, very, and extremely preterm (14). Interestingly, the level of SIgA was lower, although not significantly, in milk from smoker mothers (17).

The concentration of milk IgG is the highest during the first 3 days of lactation of term and preterm groups (27.9 ± 23.2 and 41.7 ± 17.3 mg/L, respectively); however, in the next days of lactation, from 4 to 55 days, it decreased and remained at a similar level, 16.6 ± 10.6 and 16.7 ± 8.8 mg/L, respectively regardless of the week of delivery (18). A lack of difference in the concentration of IgG in relation to the stage of lactation during the first 6 months was also demonstrated by Goonatilleke et al. (15). Similarly to SIgA, there were no differences in IgG concentrations in the milk of mothers giving birth preterm (very preterm: 16.4 ± 6.1 mg/L; moderate preterm: 20.1 ± 13.0 mg/L), regardless of the degree of prematurity (18). These values are consistent with that of about 20 mg/L given by Broadhurst et al. (19) for the same lactation period and lower than that established by Koenig et al. (20) for early colostrum of term (54 ± 37 mg/L) and preterm birth (very preterm, 76 ± 38 mg/L; moderately preterm, 47 ± 42 mg/L). On the other hand, the report of Abuidhail et al. (21) showed that the IgG concentration in milk samples derived from the 1st month of lactation was significantly lower (103 ± 41 mg/L) than that for the 4th and 6th month of lactation (133 ± 49 and 145 ± 54 mg/L, respectively), although the values were higher than previously reported.

The IgM concentration in colostrum is at a similar low level of about 14 mg/L for term and 11 mg/L for preterm birth, but the presence of IgM was found in only a small percentage of milk samples (20). Additionally, the latest reports of Goonatilleke et al. (15) showed the gradual decrease in IgM concentration from the beginning of lactation until 6 months. Similar results were presented by Abuidhail et al. (21), who showed that the IgM concentration in milk samples from the 1st month of lactation was significantly higher (103 ± 31 mg/L) than for the 4th and the 6th month of lactation (64 ± 25 and 48 ± 18 mg/L, respectively) (21). Moreover, the concentration of IgM from the 2nd to the 6th day of lactation was positively associated with overweight and obesity of the mother before pregnancy as well as with primiparity and negatively associated with smoking during pregnancy (22).

After birth, newborns are completely reliant on maternal immunoglobulins because of the immature immune system. Immunoglobulins delivered with mother's milk are crucial in shaping neonatal immunity during the first 3 months since there is a lack of functional plasma cells which are responsible for the synthesis of the newborn's IgG (11, 23). The main biological functions of SIgA include intracellular neutralization of viruses and inhibition of their transcytosis, inhibition of adhesion of pathogenic bacteria to host mucosa, and agglutination of viruses and bacteria (12, 24–26). Additionally, thanks to the interaction with intestinal M cells, SIgA takes part in the prevention of pathogenic bacteria translocation (27).

Maternal immunoglobulins, in particular, SIgA, have lasting beneficial effects on the support and regulation of the immature immune system of breastfed infants as well as on their gut microbiome. However, it should be pointed out that their specificity is related to pathogens with which the lactating mother had contact previously (28–30). Interestingly, as reported by Royle et al. (31), milk SIgA possesses an “additional binding site” for bacterial lectin receptors, i.e., sialylated and/or fucosylated glycans, besides their Fab antigen-binding sites, which constitute the link between innate and acquired immunity.

In addition to antimicrobial activity, IgG are also able to activate phagocytes, exhibit anti-inflammatory activity, and suppress abnormal inflammatory reactions in response to allergens (24). Moreover, it is suggested that sialylated and fucosylated glycans of milk IgG may play a similar role as glycans of milk SIgA (18, 32). Maternal milk IgM also takes part in the protection of newborns against pathogens via opsonization of Gram-negative bacteria (33) and is important, in addition to milk SIgA, for immune exclusion of antigens (4).

However, IgG and IgM, present in small amounts in mother's milk due to digestion in the small intestine, likely play a small role in providing passive immunity to the infant (13). Nevertheless, the latest reports of the Demers-Mathieu group showed that gastric digestion reduced IgG, but the other antibodies were not digested in the gastric contents of preterm infants and all maternal isotypes present in breast milk were detected in infant stools, of which IgA (not SIgA) was the most abundant (34).

In light of the undeniable participation of maternal milk immunoglobulins in supporting the development and the modulation of the immature immune system of newborns and infants as well as the long-term benefits of breast milk feeding, it is extremely important in promoting breastfeeding over the recommended 6 months to determine the immunoglobulin profile of human milk over the first year of lactation to provide strong scientific evidence of the existence of immune protection when antibody production by children has not yet reached levels that will allow effective protection. The lack of detailed data on the level of immunoglobulins in late lactation prompted us to investigate the concentration of skim milk SIgA, IgG, IgM, and total protein in prolonged lactation from the 1st until the 48th month. Specifically, the aim of our study was to determine whether the levels of immunoglobulins in mother's milk over the 1st year of lactation are the same as up to the 1st year and whether there are any correlations among the concentrations of particular immunoglobulins as well as with protein concentration and finally to evaluate the potential correlation of immunoglobulin concentrations with the frequency of breastfeeding during 4 years of lactation.

## Materials and Methods

### Milk Collection

Lactating women were recruited using local parenting groups that communicated via social media. One hundred sixteen breastfeeding mothers participated in the study. The characteristics of breastfeeding mothers, as well as the method of collecting and storing milk samples, have been described previously (35, 36). The mother's age, socioeconomic status, race, health status, concomitant medications, parity, mode of delivery, and frequency of breastfeeding were recorded. Milk samples were collected at the Regional Human Milk Bank in the Department of Neonatology located in the University Hospital (Wroclaw, Poland) between 8 a.m. and 2 p.m. Providing an interval of a few hours allows a greater uniformity of samples. Considering the efficiency of milk expression, an electric breast pump was used (Medela Symphony, Baar, Switzerland). Aliquots for analysis (2–3 mL) were taken immediately after complete emptying of the breast, and the whole volume of the expressed milk was gently stirred to minimize the possibility of any preanalytical fault. The milk samples for analysis were divided into four groups according to breastfeeding period: the first group up to 12 months (*N* = 26), the second from 13 to 18 months (*N* = 35), the third from 19 to 24 months (*N* = 32), and the last over 24 months (*N* = 23). For storage, the samples were aliquoted into smaller containers and frozen at −20°C. This study received ethical approval (Nr KB−65/2018) from the University Ethics Committee. Informed and written consent was provided by all the participants before sample collection.

### Sample Preparation

To obtain the skim milk fraction, samples were centrifuged at 3,500 g at 4°C for 35 min, after which milk fat and cells were separated. The samples of the aqueous phase of milk (skim milk) were kept at −20°C until determination.

### Determination of Skim Milk Protein Concentration

The total protein concentration in human skim milk samples was determined by bicinchoninic methods with the Bicinchoninic Acid Protein Assay Kit (Sigma, St. Louis, MO, USA) (37). For testing 25 μL of 12.5- and 25-fold diluted in TRIS-buffered saline (TBS, pH 7.5), skim milk samples and bovine albumin, as a standard from 0.2 to 1.0 mg/mL (SERVA, Heidelberg, German), were prepared and transferred to the wells of microtiter plates, and then 200 μL of freshly prepared bicinchoninic acid working reagent (solution of bicinchoninic acid and copper (II) sulfate in the ratio 1:50, respectively) was added. The plates were incubated at 37°C for 35 min and the absorbance was measured at 560 nm in a Stat Fax 2100 Microplate Reader (Awareness Technology Inc., Palm City, FL, USA). All skim milk samples were analyzed in duplicate.

### Determination of SIgA Concentration

The concentrations of SIgA in skim milk samples were determined by ELISA. For testing, 100 μL of 10,000-, 25,000-, and 50,000-fold diluted skim milk and human colostrum IgA standard preparations from 1.2 to 37.5 ng/100 μL (Sigma, St. Louis, MO, USA) were taken, and then the plates were incubated for 2 h at 37°C. After the washing step, TBS (pH 7.5), containing 0.2% Tween-20 as a blocking agent, was used and the plates were incubated for 1 h at 37°C and overnight at 4°C. As a detection antibody, mouse monoclonal anti-secretory component IgA antibodies (Sigma, St. Louis, MO, USA) diluted 1:10,000 in TBS containing 0.05% Tween-20 were used, and the plates were incubated for 1 h at 37°C. In the next step, horseradish peroxidase-conjugated goat anti-mouse IgG antibodies (Sigma, St. Louis, MO, USA) diluted 1:5,000 in TBS containing 0.05% Tween-20 were added to each well, and the plates were incubated for 1 h at 37°C. The reaction was developed by adding a substrate solution containing orthophenylenediamine (Calbiochem, Denmark) in 0.1 M citrate buffer, pH 5.0 with H_2_O_2_, and the plates were incubated for 30 min at room temperature in the dark. The reaction was stopped with 12.5% H_2_SO_4_, and the absorbance was measured in a Stat Fax 2100 Microplate Reader (Awareness Technology Inc., Palm City, FL, USA) at 492 nm with 630 nm as the reference filter. For all washing steps, TBS (pH 7.5) containing 0.05% Tween-20 was used.

The coefficients of variation were calculated for SIgA-ELISA, namely, 3.4 and 2.9% for intra- and inter-assay, respectively.

### Determination of IgG Concentration

As described previously (18, 32), the concentration of IgG in skim milk samples was determined by conventional ELISA. In short, F(ab')2 fragments of goat anti-human IgG (Jackson ImmunoResearch, Europe Ltd., Ely, UK) antibodies diluted 1:1,000 in TBS as a capture antibody were taken, and the plates were incubated for 2 h at 37°C. For the blocking step, TBS (pH 7.5) containing 0.5% Tween-20 was used, and the plates were incubated for 2 h at 37°C and overnight at 4°C. For testing, 100 μL of 500-, 1,000- and 2,500-fold diluted skim milk and human serum IgG as a standard preparation from 0.2 to 12.5 ng/100 μL (Jackson ImmunoResearch, Europe Ltd., Ely, UK) were taken, and the plates were incubated for 1 h at 37°C. The detection was carried out with phosphatase-labeled rabbit anti-human IgG Fcγ fragment-specific antibodies diluted 1:20,000 in TBS with 0.05% Tween-20 (Jackson ImmunoResearch, USA), and the plates were incubated for 1 h at 37°C. The reaction was developed by adding 4-nitrophenyl phosphate in diethanolamine-HCl buffer (pH 9.5) (SERVA, Heidelberg, Germany), and the plates were incubated for 15 min at 37°C. The reaction was stopped with 1 M NaOH and the absorbance was measured in a Stat Fax 2100 Microplate Reader (Awareness Technology Inc., Palm City, FL, USA) at 405 nm, with 630 nm as the reference filter. For the dilution of skim milk samples and for all washing steps, human serum IgG standard preparation and TBS (pH 7.5) containing 0.1 or 0.05% Tween-20 was used, respectively.

The coefficients of variation were calculated for IgG-ELISA, namely, 2.9 and 5.7% for intra- and inter-assay, respectively.

### Determination of IgM Concentration

The concentrations of IgM in skim milk samples were determined by conventional ELISA. In detail, AffiniPure rabbit anti-human IgM antibody as a capture antibody (Jackson ImmunoResearch, Europe Ltd., Ely, UK) diluted 1:2,000 was taken, and the plates were incubated for 2 h at 37°C. Then, the plates were incubated for 2 h at 37°C and overnight at 4°C with TBS (pH 7.5) containing 0.5% Tween-20 as a blocking agent. For testing, 100 μL of 50-, 100-, and 200-fold diluted skim milk and human IgM standard preparation from 0.39 to 6.25 ng/100 μL (Jackson ImmunoResearch, Europe Ltd., Ely, UK) were taken. In the next step, horseradish peroxidase-conjugated goat anti-human IgM antibodies (Jackson ImmunoResearch, Europe Ltd., Ely, UK) diluted 1:20,000 in TBS containing 0.05% Tween-20 were added to each well, and the plates were incubated for 1 h at 37°C. The reaction was developed by adding orthophenylenediamine (Calbiochem, Denmark) in 0.1 M citrate buffer, pH 5.0 with H_2_O_2_, and the plates were incubated for 10 min at room temperature in the dark. The reaction was stopped with 12.5% H_2_SO_4_, and the absorbance was measured in a Stat Fax 2100 Microplate Reader (Awareness Technology Inc., Palm City, FL, USA) at 492 nm, with 630 nm as the reference filter. For dilution of skim milk samples and for all washing steps, TBS (pH 7.5) containing 0.1 or 0.05% Tween-20 was used.

The coefficients of variation were calculated for IgM-ELISA, namely, 5.4 and 2.5% for intra- and inter-assay, respectively.

### Statistical Analysis

The statistical analysis was performed with TIBCO STATISTICA 13.3 (StatSoft, Inc., Tulsa, OK, USA). The results are expressed as the mean ± SD (standard deviation) and the median with 25th−75th percentiles. To compare the study population data, chi-square test was used. For analysis, non-parametric tests were used since large interindividual differences in the biochemical profile of milk are observed among lactating mothers. For the calculation of statistical significance, Kruskal-Wallis test was used. The correlations between the analyzed groups were estimated according to Spearman. A two-tailed *p*-value lower than 0.05 was considered as significant.

## Results

The detailed characteristics of the analyzed cohort of lactating mothers are shown in Table 1.

**Table 1:** Characteristics of the study population.

	**Breastfeeding ≤12 months *N* = 26 [% (*n*/*N*)]**	**Breastfeeding >12 months *N* = 90 [% (*n*/*N*)]**	**χ^2^**	***p*-value**	
Maternal age			4.2262	0.1208	
25–29	50 (13/26)	32.5 (29/89)			
30–34	42 (11/26)	44 (39/89)			
35+	8.3 (2/26)	23.5 (21/89)			
Race/ethnicity			-	-	-
White Europeans	100 (26/26)	100 (90/90)			
Socioeconomic status and education			0.3208	0.5711	
Secondary education	11.5 (3/26)	16 (14/90)			
Higher education	88.5 (23/26)	84 (76/90)			
Parity			0.9915	0.3193	
1	78 (20/24)	67 (60/90)			
2	22 (6/24)	31 (28/90)			
3	0 (0/24)	2 (2/90)			
Gender					
Male	10/23	36/61	1.061	0.3029	
Female	13/23	25/61			
Birth weight			-	-	-
Appropriate for gestational age	100 (26/26)	100 (90/90)			
Gestational age			0.8906	0.3453	
34–37	4 (1/26)	1 (1/90)			
>37	96 (25/26)	99 (89/90)			
Mode of delivery	16/22	34/65	2.0308	0.1541	
Vaginal birth	6/22	31/65			
Cesarian section					
Medicines during lactation			0.2645	0.8761	
No medications	65 (17/24)	70 (63/90)			
Thyroxine	27 (7/24)	25 (22/90)			
Others	8 (2/24)	5 (5/90)			
Maternal diet during lactation			-	-	-
Vegan/vegetarian	0 (0/24)	8 (7/90)			
Dairy-free diet	23 (6/24)	7 (6/90)			
Gluten-free diet	0 (0/24)	3 (3/90)			
Complementary foods introduction Above 6 months of life	NA	97 (87/90)	-	-	-

*-, not analyzed*.

### Concentration of Skim Milk Protein

**Figure 1:**
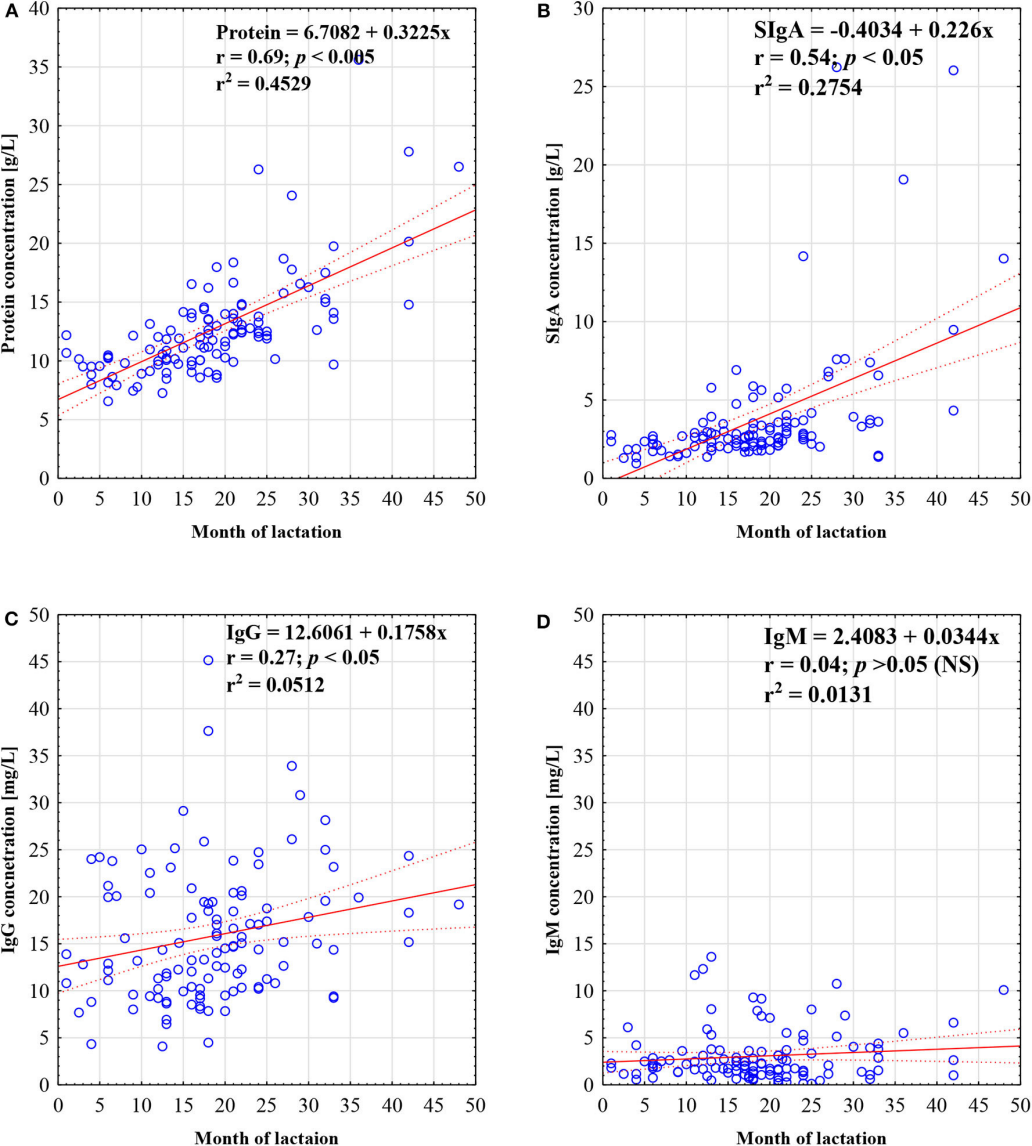
Concentration of protein **(A)**, SIgA **(B)**, IgG **(C)**, and IgM **(D)** in skim milk in prolonged lactation up to 48 months. A solid line indicates linear regression, and 95% confidence intervals are shown by dotted lines; blue circles refer to individual samples. The correlation coefficient (*r*) was calculated according to Spearman, and a *p*-value lower than 0.05 was regarded as significant. The *r*-square value is the square of the correlation coefficient. NS, not significant.

The concentration of protein in mother's skim milk showed a strong positive correlation with the duration of lactation from the 1st to the 48th month (*r* = 0.69; *p* < 0.05) (Figure 1A).

The mean concentration of protein was the lowest (9.69 ± 1.59 g/L) for the first year of lactation (1–12 months) and significantly increased in the next analyzed period of prolonged lactation to reach 11.47 ± 2.23 g/L (*p* < 0.003) in the milk group from 13 to 18 months, 13.13 ± 3.31 g/L (*p* < 0.002) in the milk group from 19 to 24 months, and finally 17.31 ± 6.20 g/L (*p* < 0.002) in lactation beyond 2 years (Table 2).

**Table 2:** Concentration of protein and immunoglobulins in skim milk in prolonged lactation.

**Skim milk immunoglobulins**	**Group**				***r* and *p-*value**
	**1–12 months *N* = 26**	**13–18 months *N* = 35**	**19–24 months *N* = 32**	**>24 months *N* = 23**	
Protein [g/L]	9.69 ± 1.59 9.63 (8.64–10.47)	11.47 ± 2.23 11.11 (9.96–13.49) *p*^a^ < 0.003	13.13 ± 3.31 12.58 (11.71–13.88) *p*^b^ < 0.002	17.31 ± 6.20 15.75 (12.62–19.75) *p*^c^ < 0.002	*r* = 0.69 *p* < 0.005
SIgA [g/L]	2.12 ± 0.62 2.15 (1.62–2.63)	2.95 ± 1.30 2.64 (2.05–3.17) *p*^a^ < 0.006	3.35 ± 2.22 2.80 (2.32–3.57)	7.55 ± 7.16 4.33 (3.31–7.63) *p*^c^ < 0.003	*r* = 0.54 *p* < 0.05
IgG [mg/L]	14.71 ± 6.18 12.85 (9.60–20.41)	14.82 ± 9.11 11.86 (8.65–19.26)	15.60 ± 4.33 15.40 (12.37–18.02)	18.95 ± 6.76 18.32 (14.37–24.35)	*r* = 0.27 *p* < 0.05
IgM [mg/L]	3.0 ± 2.89 2.22 (2.88–2.89)	2.81 ± 2.74 2.00 (1.23–2.91)	2.79 ± 2.41 1.74 (1.07–3.61)	3.82 ± 3.05 3.33 (1.16–5.51)	*r* = 0.04 (NS) *p* > 0.05

*Values are given as the mean ± SD and median and 25th−75th percentiles in parentheses. The Kruskal–Wallis test was used for statistical calculations, and a p-value lower than 0.05 was regarded as significant*.

a *Significantly different from the milk sample group of 1–12 months*.

b *Significantly different from the milk sample group of 13–18 months*.

c *Significantly different from the milk sample group of 19–24 months. NS, not significant*.

### Concentration of Skim Milk Secretory IgA

The concentration of secretory IgA in mother's skim milk showed a strong positive correlation with lactation from the 1st to the 48th month (*r* = 0.54; *p* < 0.05) (Figure 1B).

The mean concentration of SIgA was the lowest (2.12 ± 0.62 g/L) for the first analyzed group of milk, namely, from the 1st to the 12th months, and significantly increased above the 1st year of lactation, namely, in the group of milk obtained from the 13th to 18th months of lactation, it reached 2.95 ± 1.30 g/L (*p* < 0.006). In the subsequent period of lactation in milk from 19 to 24 months, the mean value of SIgA concentration was 3.35 ± 2.22 g/L and it was higher, although not significantly, in comparison to the previous group. In lactation beyond the 2nd year, the significantly (*p* < 0.003) highest concentration of SIgA was observed (7.55 ± 7.16 g/L) (Table 2).

### Concentration of Skim Milk IgG

The concentration of IgG in mother's skim milk, similar to SIgA, showed a positive correlation with lactation from the 1st to the 48th month, but the observed correlation was significantly weaker (*r* = 0.27; *p* < 0.05) (Figure 1C).

The mean concentration of IgG in mother's skim milk was the lowest (14.71 ± 6.18 mg/L) for the first year of lactation and remained at an almost unchanged level in the next analyzed period of prolonged lactation, namely, 13–18 months (14.82 ± 9.11 mg/L). Nevertheless, in the milk groups from 19 to 24 months and above 24 months, an increase, although not significant, to the values of 15.60 ± 4.33 and 18.95 ± 6.76 mg/L, respectively, was observed (Table 2).

### Concentration of Skim Milk IgM

The concentration of IgM in mother's skim milk, in contrast to the concentration of SIgA and IgG, remained stable regardless of the lactation period (*r* = 0.04; *p* > 0.05) from the 1st to the 48th month (Figure 1D).

In the first year of lactation, the concentration of IgM was 3.00 ± 2.89 mg/L and in subsequent stages it slightly decreased, although not significantly, to the value 2.81 ± 2.74 and 2.79 ± 2.41 mg/L for groups of milk from 13 to 18 and from 19 to 24 months of lactation, respectively. In lactation beyond the 2nd year, an insignificant increase of skim milk IgM concentration to 3.82 ± 3.05 mg/L was observed (Table 2).

### Correlation Among Skim Milk Total Protein and Immunoglobulins Over Prolonged Lactation

The correlations among skim milk proteins and immunoglobulins over prolonged lactation from the 1st to the 48th month are summarized in Table 3.

**Table 3:** Correlations among skim milk protein and immunoglobulins during prolonged lactation.

	**Correlation coefficient** ***r*****-value**		
	**SIgA**	**IgG**	**IgM**
Protein	*r* = 0.71 *p* < 0.05	*r* = 0.37 *p* < 0.05	*r* = 0.008 (NS) *p* > 0.05
SIgA	-	0.29 *p* < 0.05	*r* = 0.07 (NS) *p* > 0.05
IgG		-	*r* = 0.03 (NS) *p* > 0.05

*The values of r calculated according to the Spearman method correspond to the correlation between the concentrations of skim milk protein and immunoglobulins over prolonged lactation from the 1st to the 48th month. All r values are statistically significant with p < 0.05*.

*NS, not significant*.

The concentration of SIgA and IgG showed a statistically significant strong (*r* = 0.71) and weak (*r* = 0.37) positive correlation with total skim milk protein concentration over the analyzed period of prolonged lactation. Additionally, over prolonged lactation from the 1st to the 48th month, the concentration of SIgA positively correlated with the concentration of IgG (*r* = 0.29). During the analyzed period of prolonged lactation, the concentration of skim milk IgM showed no correlation with the total concentration of skim milk protein and to the concentration of SIgA and IgG.

**Figure 2:**
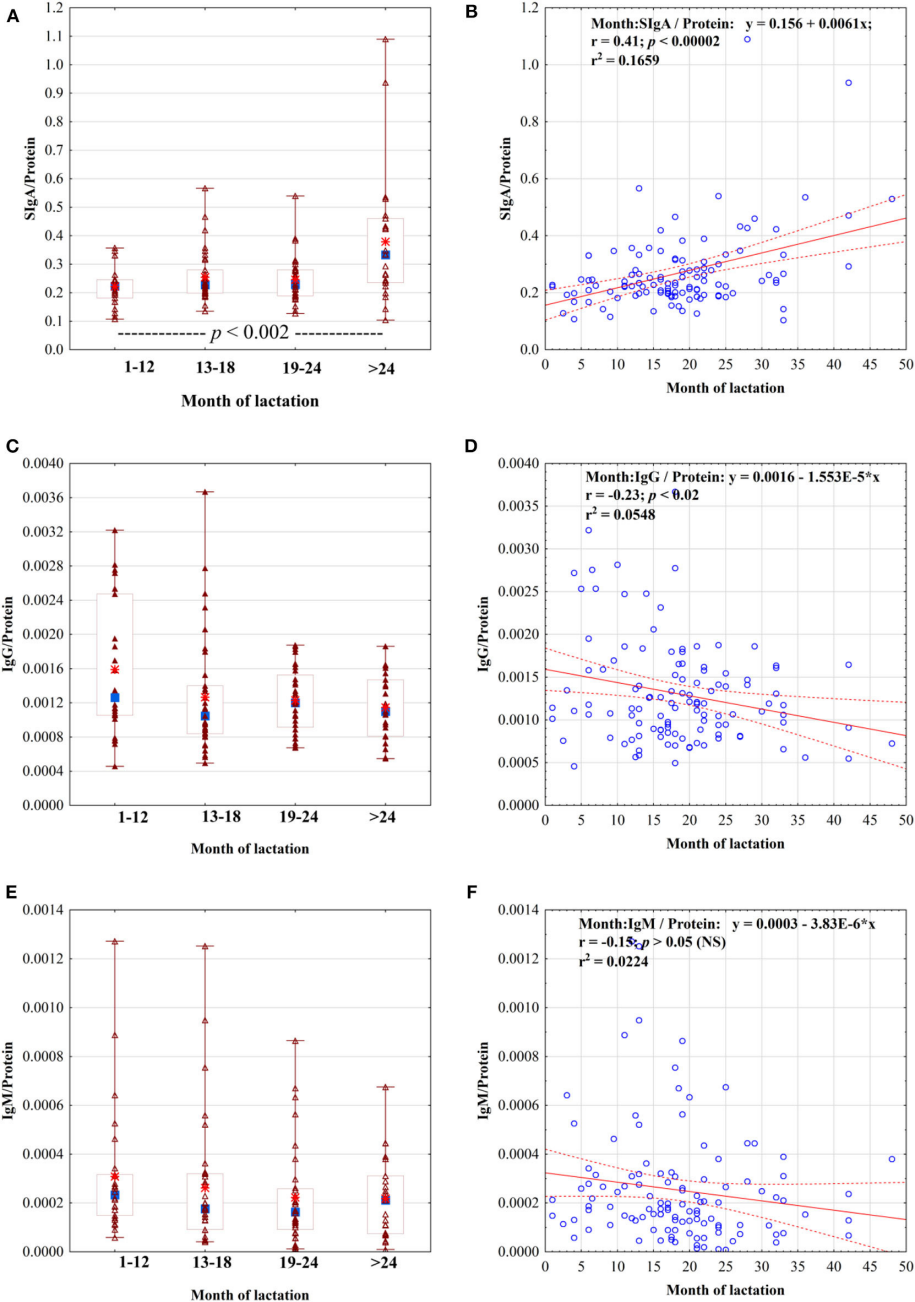
The ratio of skim milk SIgA **(A)**, IgG **(C)**, and IgM **(E)** to the protein concentration in the four groups analyzed: group 1, up to 12 months (*N* = 26); group 2, from 13 to 18 months (*N* = 35); group 3, from 19 to 24 months (*N* = 32); and group 4, over 24 months (*N* = 23), and correlation of Igs/protein ratio with 48 months of lactation: **(B)** SIgA/protein, **(D)** IgG/protein, and **(F)** IgM/protein. For **(B,D,F)**, a solid line indicates linear regression, and 95% confidence intervals are shown by dotted lines; blue circles refer to individual samples. For **(A,C,E)**, symbols: median (■), 25–75% (□), min–max (I), sample (□), and mean (*).

Additionally, the ratios of Igs to the total protein concentration in mother's skim milk were calculated (Figure 2). The ratio of SIgA to protein (SIgA/protein) remained at a similar level during the first 2 years of lactation (0.22 ± 0.07 for 1st−12th months, 0.25 ± 0.09 for 13th−18th months, and 0.25 ± 0.08 for 19th−24th months of lactation), but in the 3rd year, the SIgA/protein ratio significantly increased and reached the value of 0.38 ± 0.23 (*p* < 0.002) (Figures 2A,B). The ratios of IgG/protein and IgM/protein in mother's skim milk, in contrast to the ratio of SIgA/protein, remained stable regardless of the lactation period from the 1st to the 4th year of lactation (Figures 2C–F).

### Skim Milk Total Protein and Immunoglobulin Concentrations in Relation to Frequency of Breastfeeding

The correlations between the protein and the immunoglobulin concentrations of mother's milk with the frequency of breastfeeding (number of feedings) during the analyzed period of prolonged lactation as well as the three stages of prolonged lactation are summarized in Table 4.

**Table 4:** Relationship between number of feedings and immunoglobulin or protein concentration in breast milk during prolonged lactation.

**Immunoglobulins/protein**	**Number of feedings**^a^			
	**13–48 months**	**13–18 months**	**19–24 months**	**>24 months**
SIgA	*r* = −0.47 *p* < 0.05	*r* = −0.45 *p* < 0.05	*r* = −0.06 (NS) *p* > 0.05	*r* = −0.75 *p* < 0.05
IgG	*r* = −0.13 (NS) *p* > 0.05	*r* = 0.19 (NS) *p* > 0.05	*r* = −0.25 (NS) *p* > 0.05	*r* = −0.43 (NS) *p* > 0.05
IgM	*r* = −0.09 (NS) *p* > 0.05	*r* = 0.24 (NS) *p* > 0.05	*r* = −0.11 (NS) *p* > 0.05	*r* = −0.62 *p* < 0.05
Protein	*r* = −0.24 *p* < 0.05	*r* = 0.07 (NS) *p* > 0.05	*r* = 0.002 (NS) *p* > 0.05	*r* = −0.59 *p* < 0.05

a *The number of feedings in the analyzed groups ranged from 1 to 10 per day. The values of r calculated according to the Spearman method correspond to the correlation between the number of feedings and protein or immunoglobulin concentration in breast milk during three periods of prolonged lactation from the 13th to the 48th month. The r-values are statistically significant at p < 0.05. NS, not significant*.

**Figure 3:**
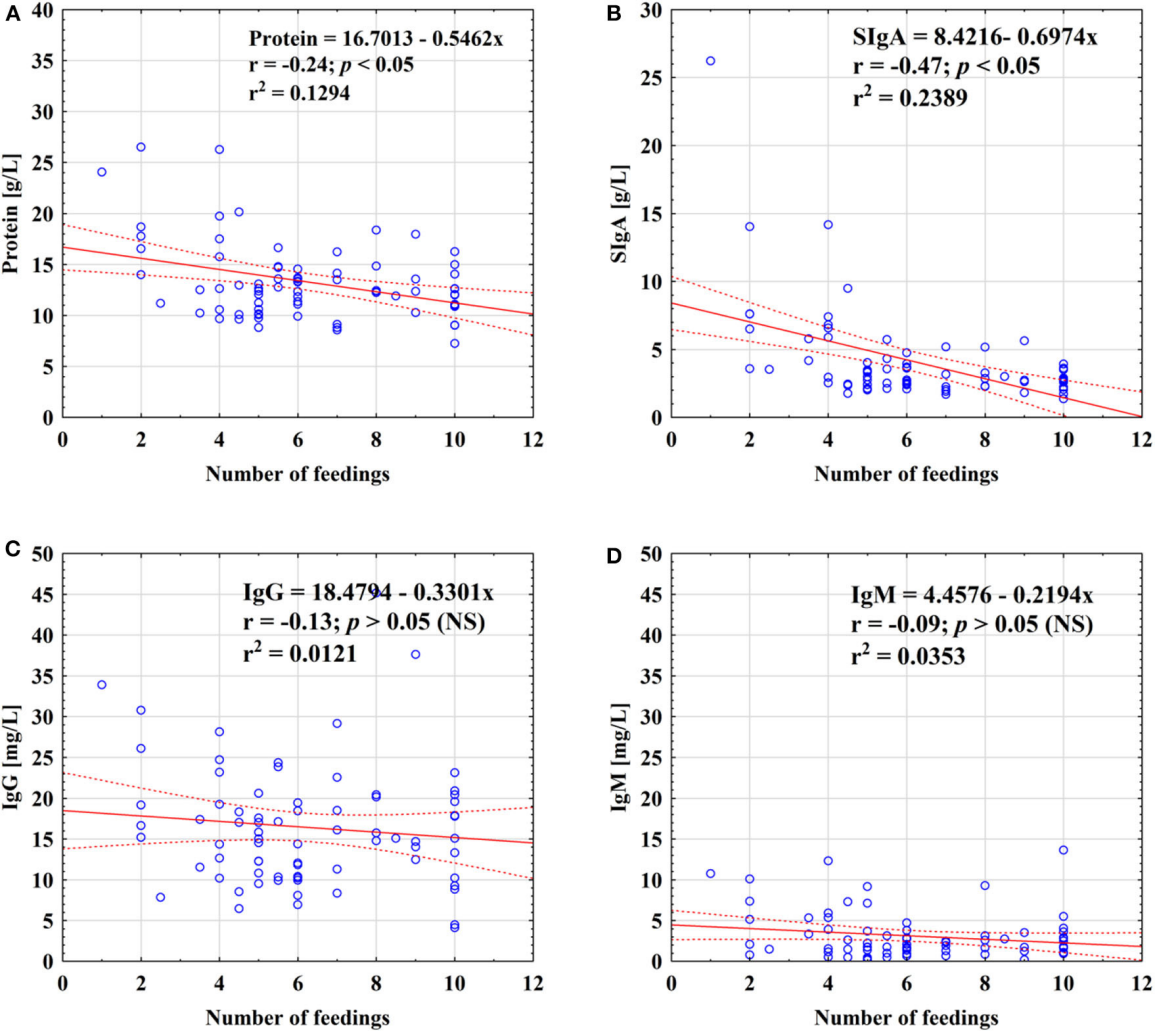
Relationship between concentration of protein **(A)**, SIgA **(B)**, IgG **(C)**, and IgM **(D)** and the number of feedings of mother's milk. A solid line indicates linear regression, and 95% confidence intervals are shown by dotted lines; blue circles refer to individual samples. NS, not significant.

For the total analyzed period of prolonged lactation from the 13th to the 48th months, negative correlations of skim milk protein (*r* = −0.24; *p* < 0.05) as well as SIgA (*r* = −0.47; *p* < 0.05) concentrations with the number of feedings were found (Figure 3). However, in the 13–18 months group, the frequency of breastfeeding was negatively correlated with the SIgA concentration (*r* = −0.45; *p* < 0.05) only (Table 4). In the subsequent lactation period, namely, 19–24 months, no statistically significant correlations were found. In contrast, after the 2nd year of lactation, the SIgA (*r* = −0.75; *p* < 0.05), IgM (*r* = −0.62; *p* < 0.05), and protein (*r* = −0.59; *p* < 0.05) concentrations were negatively correlated with the number of feedings (Table 4).

### Skim Milk Immunoglobulin Concentrations in Relation to the Mode of Delivery and the Gender of the Baby

The impact of mode of delivery, namely, vaginal birth or cesarean section, on immunoglobulin profile was analyzed in subsequent lactation periods. The SIgA and IgG concentrations were at a comparable level (*p* > 0.05) regardless of the mode of delivery in all the analyzed periods of lactation. In contrast, for IgM concentration in the first two analyzed periods of lactation, namely, for 1–12 and 13–18 months, the impact of the mode of delivery was observed. In the first year of lactation, the IgM concentration was lower in the “vaginal birth” group than in the “cesarean section” group [2.1 mg/L (*N* = 18) and 4.0 mg/L (*N* = 7) (*p*<0.03), respectively], while in the next group from the 13th to the 18th month, the IgM concentration was higher in the “vaginal birth” group than in the “cesarean section” group [3.3 mg/L (*N* = 19) and 1.6 mg/L (*N* = 11) (*p* < 0.02), respectively]. However, the amount of samples in the analyzed groups was relatively low.

The impact of gender of the baby on SIgA, IgG, and IgM concentrations was analyzed in subsequent lactation periods; however, no statistically significant differences were found (*p* > 0.05 for all immunoglobulins).

## Discussion

To the best of our knowledge, despite the undisputed importance of the immunological characterization of mother's milk at different stages of lactation, a detailed study analyzing the immunoglobulin profile in prolonged lactation up to the 4th year has not been attempted. For this purpose, sensitive methods such as ELISA with very specific antibodies adopted to meet the requirements of working with milk samples, was used. Until now, particular immunoglobulin profile studies have been limited usually up to 6 months of lactation (14, 20) only, or if it was studied for a longer period of lactation, a small number of milk samples were analyzed (15–17, 21).

During the first 3 months of life, the infant has at his disposal only maternal-derived immunoglobulins, delivered during pregnancy and breastfeeding, which are crucial for shaping and modulation of his immunity. Maternal milk immunoglobulins, due to the passive immunity transfer, are delivered to the gastrointestinal tract and participate in homeostatic mechanisms in the neonatal gut (23). Moreover, the latest reports confirmed that milk SIgA modulates the interactions between microbiota and the infant's gut and is essential for the prevention of necrotizing enterocolitis development (38).

The first 3 years of life are crucial for normal physiological and mental development. However, the immunological system is not fully mature at this age, resulting in the risk of frequent and severe infections. Long breastfeeding may provide significant support at this crucial moment in a child's life (39). Passive immunity with maternal milk antibodies may provide infant and child protection since they could neutralize pathogens already within the mammary gland, then in the infant's nasopharynx, and during swallowing in the oropharynx and hypopharynx (34).

In our study, the protein and SIgA concentrations in breast milk showed a strong positive correlation with the duration of lactation from the 1st to the 48th month. Among the four analyzed groups, namely, 1–12, 13–18, 19–24, and over 24 months, the mean concentration of SIgA was the lowest in breast milk from the 1st to the 12th month of lactation and significantly increased above the 1st year of lactation to reach the highest concentration of SIgA after the 2nd year. The concentration of IgG, similar to SIgA, showed a positive correlation with duration of lactation over 4 years. In the milk groups 19–24 and >24 months, an increase, albeit not significant, was observed. The concentration of IgM remained stable regardless of the lactation period. Over prolonged lactation, the concentration of SIgA positively correlated with the IgG concentration. We assessed the correlations among concentrations of particular immunoglobulins as well as with the protein concentration. The ratio of SIgA to protein (SIgA/protein) in mother's skim milk remained at a similar level during the first 2 years of lactation, but in the 3rd year the SIgA/protein ratio significantly increased. In contrast, the ratio of IgG to protein (IgG/protein) decreased over lactation from the 1st to the 48th month, but the ratio of IgM to protein (IgM/protein) was at the same level regardless of the period of prolonged lactation.

Our research shows that there is a high concentration of immunoglobulins in milk from prolonged lactation. Not only the frequency of feeding but also the way of feeding may affect the concentration of immunoglobulins. Abuidhail et al. (21) showed that the type of feeding has an impact on IgG concentration, namely, in exclusively breastfeeding mothers' milk the concentration of IgG was higher than in non-exclusively breastfeeding mothers' milk, but the level of IgM was not affected (21). Abuidhail et al. (21) also suggested that more time during breastfeeding and a greater volume of milk given to the babies ensure more immunoglobulin IgG and IgM because a greater breast milk volume contains more Igs. However, those results relate to the first 6 months of life.

Taking into account a previous study on prolonged lactation, after 24 months, lactation changes significantly (35, 36). After the 2nd year of lactation, the SIgA, IgM, and protein concentrations were negatively correlated with the number of feedings, and their concentrations were rising in comparison to those during the earlier period of lactation. It may be related to the reduced milk production, which decreases with the rare frequency of feeding (40). Infants eat more solids so that the volume of milk consumed is significantly less than in the first 6 months of life. The measurement of the actual amount of compounds delivered to the breastfed babies is possible (41); nevertheless, it was not measured during this study.

From a clinical point of view, mother's milk is the preferred nutrition for preterm infants (42). The highly specialized care of premature newborns includes personalization of nutrition, i.e., optimization of protein, fat, and lactose content. In our opinion, it is highly reasonable to characterize the “immunological status” of milk, which is delivered to milk banks by mothers at various stages of lactation (so-called early and late). Donor human milk (DHM) can supplement the supply of maternal breast milk when it is insufficient or provide the preferred alternative when the mother is not breastfeeding (43). Accepting breast milk from donors beyond 1 year postpartum may be a potential strategy for increasing the immunological status of donor milk. Studying the composition of such dynamic fluid as breast milk can be challenging. However, improving the understanding of the biology of breast milk as well as improving the immunological status of donor human milk for preterm infants is crucial to provide health conditions and reduce the risk of occurrence of civilization diseases such as obesity and allergies. Also, exactly how the composition of breast milk alters and the downstream effects this may have on subsequent adult health will be of great interest in regard to the programming of human metabolism during the first few years of life.

Milk of prolonged lactation is characterized by increased concentrations of fat, protein, and energy, becoming the most caloric milk during lactation (35). The high immunological potential of human milk during prolonged lactation also results in its high value. Evidence of increasing of SIgA and IgG levels and additionally the stable level of IgM during prolonged lactation provides a strong additional argument for allowing non-weaning because even if the milk from late lactation does not fully meet the energy and nutritional needs of the baby, the supply of immunological factors is beyond doubt. Based on the obtained results, we can speculate that breast milk from prolonged lactation has the potential to be used as donor milk; however, further detailed studies are needed. The use of milk from prolonged lactation gives an opportunity to concentrate a higher number of human milk components in order to improve the nutritional and the immunological status of DHM—especially due to the fact that the synthesis of IgM, similar to SIgA and IgG, by infants, in particular during early life, is not sufficient.

On the other hand, it should also be mentioned that the donor milk in a milk bank must be subject to procedures which are aimed at providing microbiological safety. Milk immunoglobulins are at least partially preserved after standard procedures of milk processing, but the effect is different for the analyzed classes of immunoglobulins. According to Escuder-Vieco and coworkers (44), the most thermostable immunoglobulin is IgG, showing the highest preservation rate (87–101%), followed by SIgA (concentration reduction of 12–46%), and the least IgM (concentration reduction of 27–75%), whereas high-pressure processing of breast milk had no or a small effect on Igs concentration (45, 46). However, the available data can differ. Adhisivam et al. present results that Holder pasteurization decreased IgA by 30% and IgG by 60% (47).

The strength of our study is the determination of the immunoglobulin profile in the 4th year of lactation since such determination has not been performed previously in such a long lactation period. Additionally, the analysis of milk samples from more than 20 mothers in each group according to the duration of lactation was performed. Moreover, in contrast to the latest reports of Klein et al. (48), who adopted triple–quadrupole time-of-flight mass spectrometry for immunoglobulin determinations, we used highly specific immunochemical ELISA methods, which we believe are better because they are cheaper, faster, more accessible, and can be routinely used in labs in milk banks, after appropriate adaptation, to characterize the immunological status of donor milk. The limitations of our study are its being a single-center, non-randomized controlled study and there being no short- and long-term health follow-up of the infants of this study.

Additionally, since Ruiz et al. (49) reported geographical variation in milk immune factor concentrations, namely, IgA, IgG, and IgM among others, the comparison/verification of our results with the results obtained for remote geographical areas using the same immunological methods and milk samples collected at the same stages of prolonged lactation is necessary (49).

## Conclusions

Our research shows that there is a high concentration of immunoglobulins in milk from late lactation. Supporting breastfeeding even after introducing solid foods should, therefore, be one of the overarching goals in the protection of public health and prevention of infections in infancy.

In view of the high concentration of immunologically important compounds present in human milk, prolonged lactation should be strongly supported.

Therefore, it is important to consider when making recommendations that not even the number of feeds per day but breastfeeding, in general, should be continued for as long as possible that the mother and the baby wish to as supplement and support for the maturing immune system of the baby.

## Data Availability Statement

All datasets generated for this study are included in the article.

## Ethics Statement

The studies involving human participants were reviewed and approved by The Bioethics Committee of the Wrocław Medical University. The patients/participants provided their written informed consent to participate in this study.

## Author Contributions

MC-Ł, JL-K, BK-O, and MO-P contributed to the conception of the study. JL-K and MO-P contributed to the design of the study. MC-Ł and JL-K organized the database. JL-K performed the statistical analysis. MC-Ł, JL-K, and MO-P wrote the first draft of the manuscript and the sections of the manuscript. All the authors contributed to manuscript revision and read and approved the submitted version.

## Conflict of Interest

The authors declare that the research was conducted in the absence of any commercial or financial relationships that could be construed as a potential conflict of interest.
